# An Integrated Peptide-Antigen Microarray on Plasmonic Gold Films for Sensitive Human Antibody Profiling

**DOI:** 10.1371/journal.pone.0071043

**Published:** 2013-07-29

**Authors:** Bo Zhang, Justin A. Jarrell, Jordan V. Price, Scott M. Tabakman, Yanguang Li, Ming Gong, Guosong Hong, Ju Feng, Paul J. Utz, Hongjie Dai

**Affiliations:** 1 Department of Chemistry, Stanford University, Stanford, California, United States of America; 2 Division of Immunology and Rheumatology, Department of Medicine, Stanford University School of Medicine, Stanford, California, United States of America; 3 Institute for Immunity, Transplantation and Infection, Stanford University School of Medicine, Stanford, California, United States of America; Weizmann Institute of Science, Israel

## Abstract

High-throughput screening for interactions of peptides with a variety of antibody targets could greatly facilitate proteomic analysis for epitope mapping, enzyme profiling, drug discovery and biomarker identification. Peptide microarrays are suited for such undertaking because of their high-throughput capability. However, existing peptide microarrays lack the sensitivity needed for detecting low abundance proteins or low affinity peptide-protein interactions. This work presents a new peptide microarray platform constructed on nanostructured plasmonic gold substrates capable of metal enhanced NIR fluorescence enhancement (NIR-FE) by hundreds of folds for screening peptide-antibody interactions with ultrahigh sensitivity. Further, an integrated histone peptide and whole antigen array is developed on the same plasmonic gold chip for profiling human antibodies in the sera of systemic lupus erythematosus (SLE) patients, revealing that collectively a panel of biomarkers against unmodified and post-translationally modified histone peptides and several whole antigens allow more accurate differentiation of SLE patients from healthy individuals than profiling biomarkers against peptides or whole antigens alone.

## Introduction

Proteomics research has focused on characterizing the structures and functions of proteins and peptides, the basic functional molecules in biological systems, affording valuable information for understanding fundamental biological processes and developing clinical applications [1]. Peptide mapping for immunogenic epitopes of whole proteins could lead to new biomarkers for disease diagnosis, prognosis and monitoring, and more effective treatment and vaccination approaches [2]–[5]. Furthermore, the identification of peptide substrates for enzyme reactivity and ligand binding could afford understanding of cellular functions, disease mechanisms and development of therapeutic strategies [6]–[9].

High-throughput screening of the reactivity of large numbers of peptides towards protein targets has been performed using various techniques, including peptide microarrays, which are especially well-suited for screening biomolecules interactions in parallel, given their high-throughput capability [10]. Through fabrication of a matrix of uniquely addressable “spots” onto solid substrate with each spot containing a unique type of peptide molecules, a multiplexed peptide array can be formed, useful for measuring a variety of protein-peptide binding events. Peptide microarrays have been employed for the identification of antibody reactivity for diagnostic applications, [11] recognition of kinase-substrate activity for drug development, [9], [12] and discovery of peptide-cell adhesion for investigating cell-cell communications [13].

Previous approaches to peptide microarrays include *in situ* synthesis of peptides directly onto substrates and robotic spotting and immobilization of pre-synthesized peptide onto microarray chips [14]. Cellulose membranes have also been used as substrates in the well-known SPOT peptide array technology, in which parallel amino acid coupling was used to synthesize unique peptides in different regions of the membrane [15]. Price *et al.* recently developed an *on silico* synthesized peptide microarray for peptide screening down to single amino acid resolution [16]. While these technologies have advanced the state of the art, SPOT assays are limited in sensitivity and quantification capability, [17] and direct synthesis on silicon substrates is a time-consuming process, taking up to three months or more. Alternatively, several types of surface chemistry have been developed for chemical immobilization of pre-synthesized peptide molecules on glass or other substrates through robotic printing. Compared to *in situ* synthesis method, spotted peptide arrays can be fabricated rapidly using pre-synthesized and well characterized peptides, as was demonstrated in the human epigenome peptide microarray platform (HEMP) [18]. Immobilization of peptides on various substrates takes advantage of covalent chemical modification of glass and gold surfaces through silane chemistry or thiol chemistry [13], [19]–[24]. Porous nitrocellulose substrates are well suited for protein microarrays with high loading capability through physical entrapment; however, nitrocellulose has low and variable binding efficiency for small molecules and is not suitable for peptide array applications [17], [25].

Due to the relatively low peptide-target binding affinity and a broad span over five orders of magnitude, [19], [26] peptide arrays lack sensitivity in detecting low affinity peptide-protein pairs. The low limit of detection (LOD) is subpar compared to peptide based enzyme linked immunosorbent assay (ELISA) and radio immunoassay (RIA) [5], [27]–[29]. It is highly desirable to develop high throughput multiplexed detection of peptide-protein interactions with increased sensitivity and broader dynamic range than is possible with current microarrays.

Here, we present a new peptide microarray platform on non-continuous, nanostructured plasmonic gold films with enhanced NIR fluorescence detection for vastly improving the sensitivity of high-throughput peptide-antibody screening. The gold platform utilizes spontaneously adsorbed avidin for immobilization of biotin-conjugated peptides and biotinylated branched PEG stars to minimize non-specific binding (NSB) background signal. A proof of concept peptide array composed of biotinylated unmodified and post-translationally modified histone peptides was first demonstrated on gold for detecting commercial antibodies with three orders of magnitude improvement in sensitivity over peptide arrays on glass, with the LOD down to the 10 femto-molar (pg/mL) range. The high sensitivity of the peptide array on gold allowed integration with antigen array on the same chip for the first time for simultaneous probing of peptide-antibody and antigen-antibody interactions over a broad dynamic range. Unmodified and post-translationally modified histone peptides together with whole antigens were arrayed on a plasmonic gold substrate for sensitive profiling of antibodies in the sera of systemic lupus erythematosus (SLE) patients vs. healthy controls. A combined set of peptides and antigens was found to give more accurate differentiation of SLE sera from normal sera than peptides or antigens alone, opening the possibility of gold supported peptide-antigen arrays for disease diagnostics.

## Results

**Figure 1:**
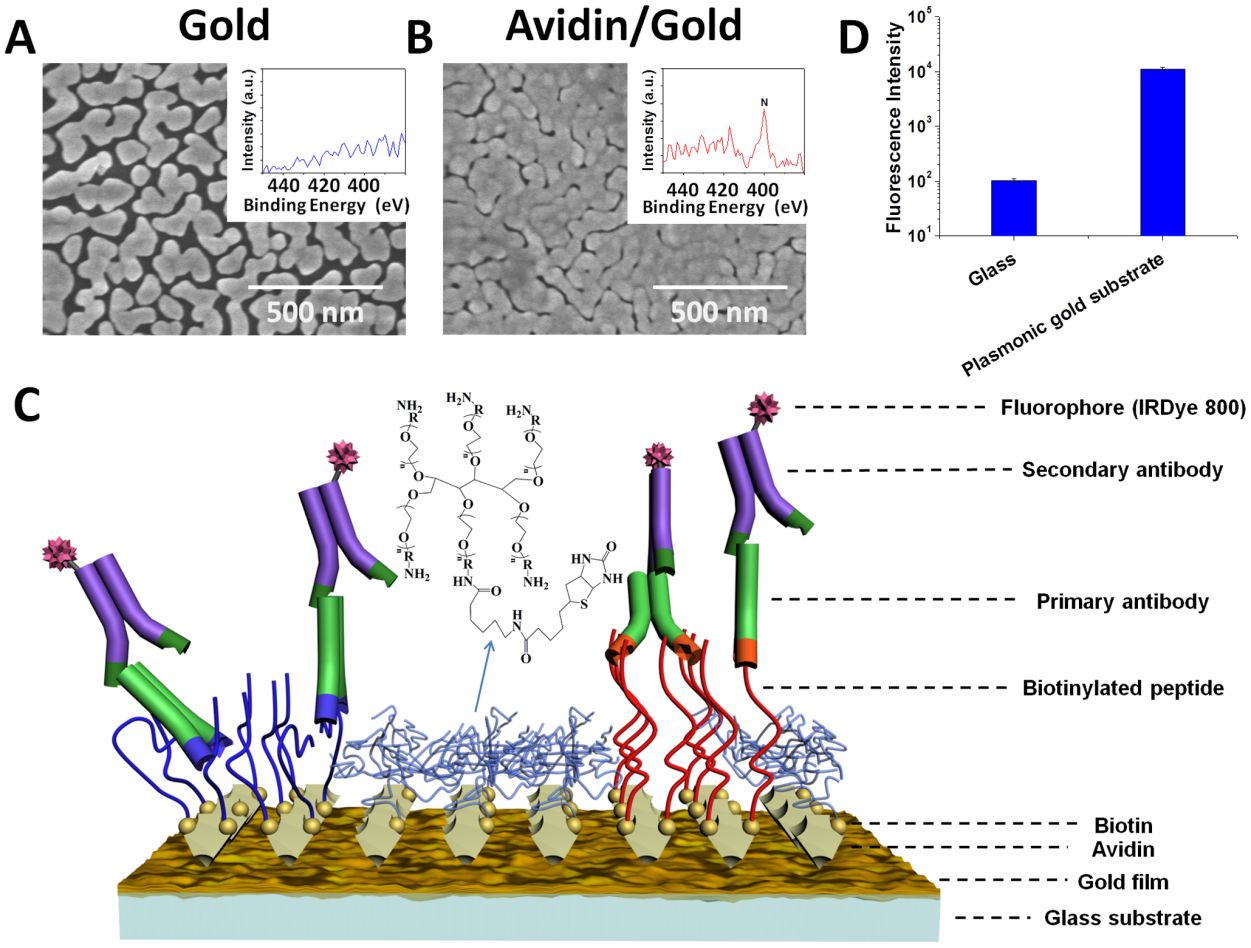
Plasmonic gold films for peptide microarrays. A) Scanning electron microscopy (SEM) of an as-made plasmonic gold film containing nano-islands formed on a glass substrate. Inset: an X-ray photoelectron spectrum (XPS) of the film. B) SEM of a plasmonic gold film coated with a layer of avidin proteins (avidin coating caused blurred features of the underlying gold nano-islands). Inset, an XPS spectrum taken on the avidin coated gold substrate showing the presence of nitrogen (in the avidin) over the film. C) A schematic drawing of a multiplexed peptide array (two peptides are shown, red and blue; peptide spots are surrounded by PEG-star coating) on gold for antibody screening based on a 3-layer assay with a NIR fluorophore reporter. D) Intensity of IRDye800 fluorescence (emission ∼ 800 nm) measured on a layer of IRDye800 labeled avidin coated on glass vs. on a plamonic gold film. A ∼100 fold fluorescence enhancement was observed on gold.

The synthesis of plasmonic gold substrate containing densely packed Au nano-islands (Figure 1A) capable of enhancing near infrared fluorophores was described previously [30]–[35]. In a pH  = 7.4 buffer solution, we found that avidin spontaneously absorbed onto the plasmonic gold films to form a uniform layer, attributed to strong electrostatic interactions with gold since in a pH  = 7.4 solution avidin was highly positively charged with an isoelectric point of pI ∼10.5. Scanning electron microscopy (SEM) imaging (Figure 1B) revealed a dense avidin coating layer on gold films. X-ray photoelectron spectroscopy (XPS) revealed the appearance of a nitrogen peak corresponding to the N (1 s) binding energy of ∼400 eV in the gold film after avidin adsorption (Figure 1B inset), confirming avidin (a nitrogen rich protein) coating on gold. Avidin proteins labeled with IRDye800 (NIR emission peak ∼800 nm) were also found to spontaneously adsorb on Au films and glass slides. We observed a NIR fluorescence enhancement of ∼100-fold for IRDy800-avidin adsorbed on Au vs. on glass (Figure 1D), while XPS detected similar N levels in the range of 6–9% (corresponding to similar avidin density) on the two substrates.

The NIR fluorescence enhancement phenomenon [30]–[34] served as the basis for our fluorescence enhanced peptide microarrays on plasmonic gold substrates. The emission of fluorophores positioned in proximity to plasmonic metal nanostructures can be enhanced due to amplified excitation electric fields between metallic nanogaps, and increased radiative decay rates of excited states due to resonance coupling between surface plasmonic modes and fluorescent emission dipoles [30]–[34], [36], [37]. Note that in most cases, fluorescence was quenched on metal films (such as continuous gold films commonly made by vapor deposition) without suitable nanostructures for electrical field enhancement or plamonic modes in resonance with fluorescence emission of the fluorophore [38], [39]. Maximum fluorescence enhancement was achieved in our case by synthetically optimizing the nanoscale gaps (in the 10–100 nm range) in the plasmonic gold films (Figure 1A) and tuning of the average size of the gold islands (in the 100–200 nm range) such that the plasmonic peaks of the gold film overlap with the NIR fluorophore emission [32]. The resulting ∼100-fold enhancement of NIR fluorophores on our gold films is an enabling factor for highly sensitive peptide microarrays. Also, detection in the NIR window is beneficial due to reduced autofluorescence background compared to the visible spectral window [40]. Note that the dependence of NIR fluorescence enhancement on the number of protein layers was found to be relatively small over multi-layer (1- to 3-layer) protein structures on plasmonic gold (Figure S1), suggesting high fluorescence enhancement achievable for sandwich type of assays on gold.

With the avidin coated plasmonic gold substrate, we performed multiplexed peptide immobilization by robotic spotting of biotinylated small peptide molecules to form a peptide array (Figure 1C). Strong biotin-avidin binding allowed for peptides efficiently immobilized onto plasmonic substrates in a multiplexed manner. The one step coating of Au with avidin was simple and highly effective for peptide microarray construction. To minimize non-specific binding (NSB) effects and reduce background signal and false positive signals, we synthesized a biotinylated, six-branched polyethylene glycol (PEG) star for blocking by immersing the peptide immobilized microarray into a biotinylated branched PEG star (see Figure 1C and Methods) solution. This step passivated the avidin coating layer on gold surrounding the peptide spots with PEG stars (Figure 1C). The use of PEG star cushion layers as a blocking layer for peptide microarrays was found important to minimize background signals (compared to linear PEG and traditional biotin blocking, Figure S2) for measuring human serum samples with complex compositions containing abundant non-specific antibodies.

**Figure 2:**
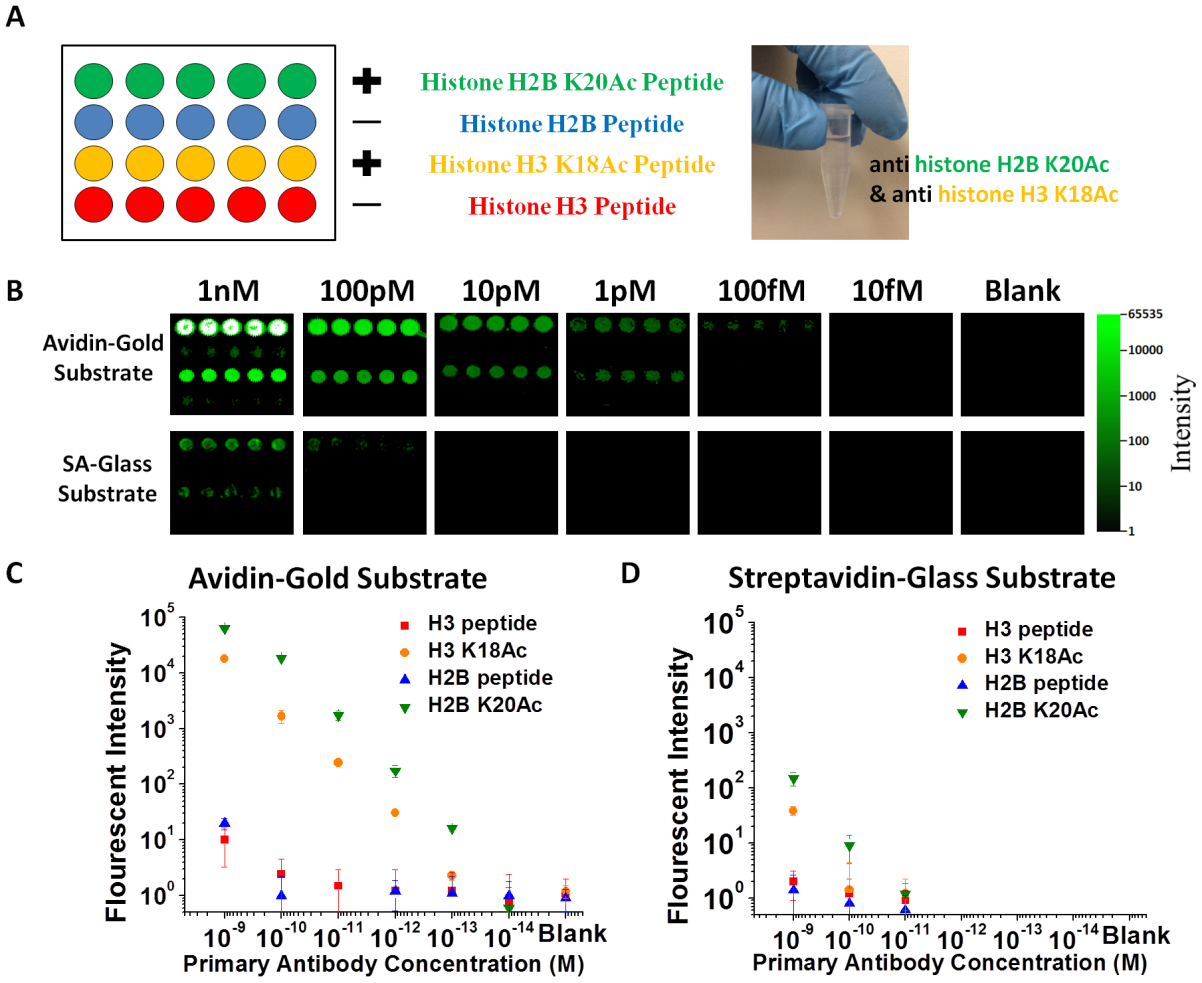
Multiplexed probing of peptide-antibody interaction on plasmonic gold vs. **glass.** A) Schematic of a 4-plex histone peptide microarray containing unmodified and acetylated Histone H2B and H3 peptides immobilized on gold plasmonic and glass substrates for interacting with a mixture of 2 commercial antibodies against the acetylated H2B and acetylated H3 peptides in a solution (right photo). B) Top panel: fluorescence images of histone peptide microarrays on gold plasmonic substrate probed in titration with commercial antibodies targeting acetylated H2B and H3 peptide. Bottom Panel: identical peptide microarrays on streptavidin coated glass slides probed with the same commercial antibodies as top panel. C) and D) are calibration curves for antibody quantification generated by averaging the integrated fluorescence intensity of secondary antibody-IRDye800 emission over the 5 duplicate microarray spots for each of the histone peptide at various concentrations of histone antibodies on plasmonic gold slide and streptavidin-glass slide. Error bars represent the standard deviation of the mean over the 5 duplicate assay features.

Having generated and optimized peptide microarrays on gold, we probed histone peptide-antibody interactions using fetal bovine serum (FBS) solutions spiked with commercial antibodies. Histones are major components of chromatin and also play important biological roles in gene regulation. Post-translational modifications of the N-terminal domains (tail regions) of histone protein/peptides, such as acetylation, phosphorylation, and citrullination are involved in disease development and serve as epigenetic markers [41], [42]. To establish the use of peptide arrays on plasmonic substrates for specific peptide-antibody binding, we designed a 4-plex peptide array (Figure 2A) containing unmodified histone H2B peptide comprised of 21 amino acid at the tail region of histone H2B protein, H2B peptide with the 20^th^ lysine residue acetylated (H2B K20Ac), histone H3 peptide composed of 21 amino acid at the tail region of histone H3 protein, and H3 peptide with the 18^th^ lysine acetylated (H3 K18Ac).

Serial dilutions of a mixture of two commercial antibodies against H2B K20Ac and H3 K18Ac each at 1 nM to 10 fM concentration were used to probe the histone peptide microarray followed by secondary antibody-IRDye800 detection (Figure 2B). We observed hundreds of fold NIR fluorescence enhancement on our avidin coated gold (Figure 2B top panel) when compared with commercial streptavidin coated glass (Figure 2B bottom panel), accompanied by a 3-order of magnitude increase in dynamic range and sensitivity with signal linearity down to 10 fM (Figure 2C). The specificity of detection was high, but a weak signal on unmodified histone peptide was observed with the high, 1 nM incubation concentration of antibody solution (Figure 2C blue symbols for signals at 1 nM), which was attributed to weak cross reactivity of the commercial antibody. This result suggested that a single-site post-translational modification of histone peptide could indeed significantly alter peptide-antibody binding, detectable on plasmonic gold substrate down to ∼10 fM protein concentrations in spiked serum samples.

Next, we investigated plasmonic gold peptide-antigen microarrays for profiling autoantibodies in human serum samples. Systemic lupus erythematosus (SLE) is the second most common rheumatic autoimmune disease in the United States and diagnosis is complex and includes clinical and laboratory criteria [43]. Antinuclear antibody (ANA) assay and further testing of anti-dsDNA and anti-Smith antibodies are part of the laboratory diagnostic evaluation for SLE. Measurement of serum autoantibodies reactive against unmodified peptide probes in 96-well plates (ELISA), or biotinylated peptides immobilized on streptavidin coated glass slides, indicated that circulating levels of serum autoantibodies targeting modified and unmodified histone proteins and correlated positively with SLE disease [41], [44], [45]. Thus, developing an assay for screening large numbers of histone peptides (unmodified and modified) for identification of peptide biomarkers for SLE diagnosis is of importance to SLE research and potential clinical translation.

**Figure 3:**
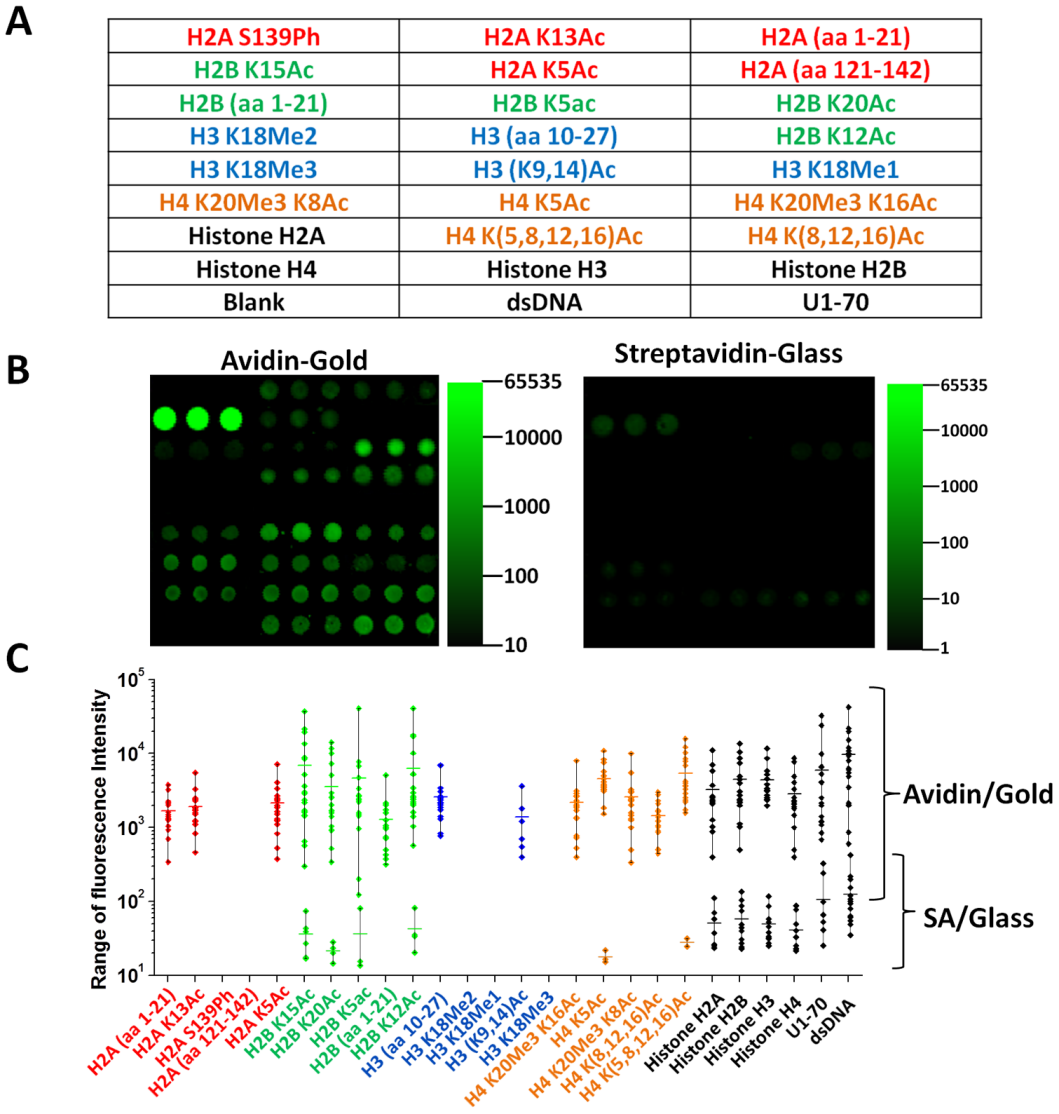
An integrated peptide-antigen microarray for profiling serum antibodies of 20 patients with systemic lupus erythematosus (SLE). A) Peptide-antigen microarray layout including modified and unmodified histone H2A, H2B, H3, H4 peptides as well as whole antigens. Ac: acetylated; aa: amino acid; Me1: methylated; Me2: dimethylated; Me3: trimethylated; Ph: phosphorylated; K: Lysine; S: Serine. Number indicates amino acid position from the N-terminus of its corresponding histone proteins. Peptide sequences are listed in Table S1. B) Microarray imaging results of a SLE patient serum probed on a plasmonic gold substrate (left) vs. on a commercial streptavidin-glass substrate (right) shown in the same IRDye800 fluorescence intensity scale. C) Dynamic range of fluorescence signal reading among 20 SLE patients measured on each peptide and antigen spots on avidin/gold slide (upper panel) and commercial streptavidin/glass slide (lower panel). Each dot represents a fluorescence intensity measured on a peptide or antigen spot shown along the x-axis in the serum of each of the 20 SLE patients. A dot is drawn only when the fluorescence intensity is above the background by 2 standard deviation of the background signal.

We constructed peptide-antigen microarrays on plasmonic gold and glass substrates with a panel of 20 histone peptides as well as 6 whole antigens (H2A, H2B, H3 and H4, dsDNA, and small nuclear ribonucleoprotein U1–70) to characterize autoantibodies in the sera of 20 SLE patients (Figure 3A, Table S1). Whole antigens were found to bind to the avidin coated gold substrate upon spotting (likely through electrostatic interactions with the highly charged avidin) without the need for biotinylation. Histone peptide and antigen arrays on plasmonic gold substrates were probed with serum samples collected from clinically confirmed SLE patients diluted 200 fold in PBS containing 20% FBS. We detected human antibodies binding to a sub-set of histone peptides and whole antigens on the plasmonic gold substrate, and the fluorescence signals were much higher (>2 orders of magnitude) than the corresponding features on the glass substrate (Figure 3B and 3C). Also, the fluorescence intensities of peptide spots on gold spanned a much broader range than the corresponding spots on glass (Figure 3C), suggesting better performance for differentiating peptide-antibody interactions on plasmonic gold substrates. On the streptavidin-glass substrate, antibody reactivity to whole antigen spots was observed, but fewer peptides were found to react with the antibodies in sera derived from SLE patients than were detected on gold (Figure 3B and 3C) due to the low peptide array sensitivity on glass. Only the 4 acetylated H2B peptides (not the unmodified H2B peptide) were observed to react with SLE sera antibodies on glass (Figure 3C lower green symbols).

**Figure 4:**
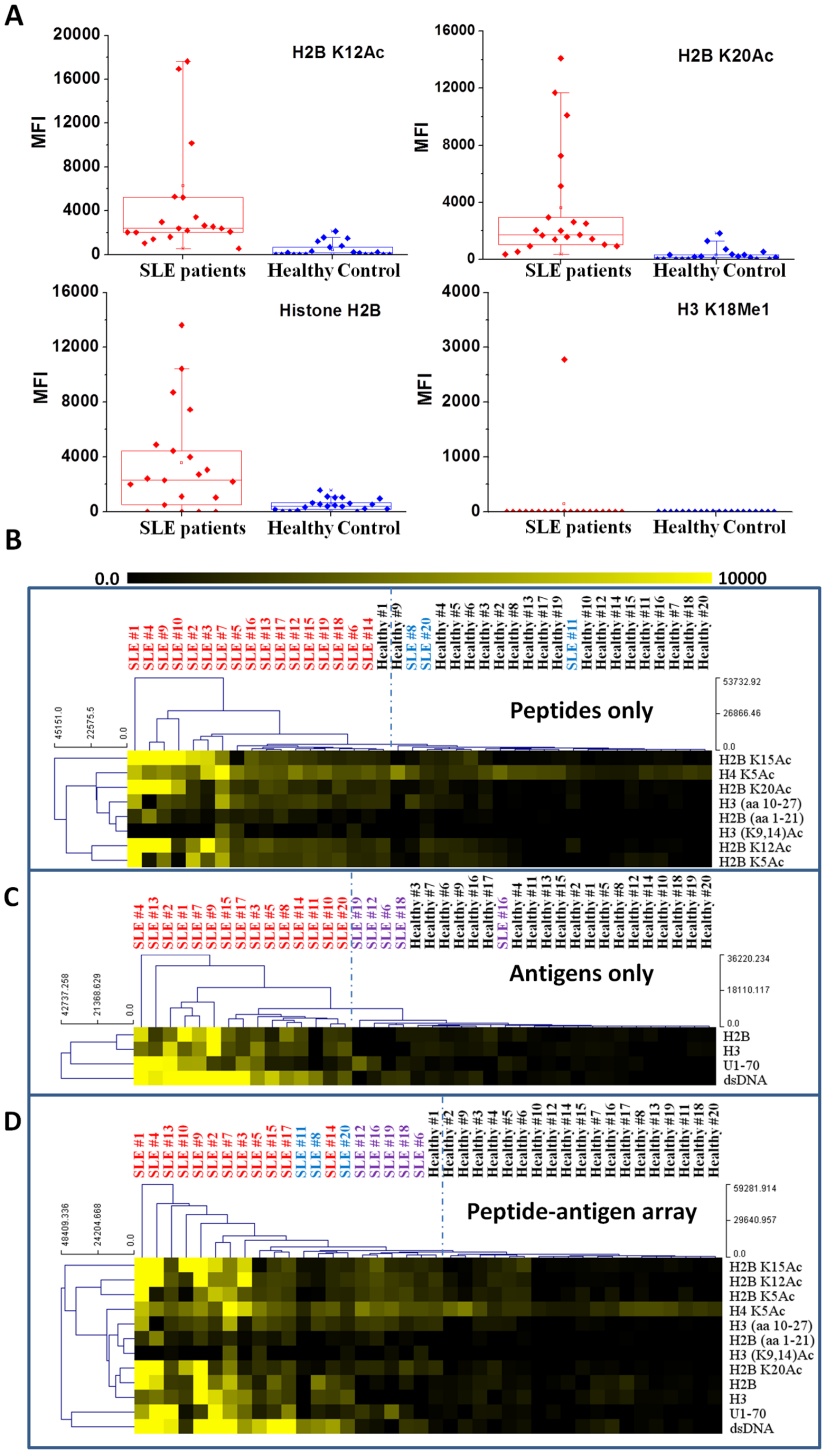
Peptide-antigen microarrays on plasmonic gold substrate for profiling antibodies in serum samples of SLE patients and healthy individuals. A) Box plot of serum IgG antibody reactivity against several peptides and a whole antigen for 20 SLE patients and 20 healthy controls. Acetylated histone H2B peptides were found to be able to differentiate SLE patients and healthy controls (top box plots) together with whole H2B protein (bottom left box plot). While the H3 peptide with the 18th lysine methylated were found not capable of telling SLE patient from healthy control (bottom right box plot). B–D) Heatmaps displaying antibody reactivity to (B) histone peptides only, (C) whole antigens only, and (D) a combination of histone peptides and whole antigens that are identified capable of differentiating SLE patients and healthy controls with false discovery rate (q value) <0.001. The dashed lines are drawn to highlight the separation of SLE and healthy groups identified by using the average linkage Euclidean distance hierarchical clustering method. Color intensity of each grid in the heatmap reflected mean fluorescence intensity of corresponding peptide or antigen spot on the microarray for each SLE patient or healthy individual. In (B) and (C), several SLE patients (labeled in blue and purple color) are misplaced in the healthy group. These patients are grouped in the SLE side in (D) that profiles antibodies against both peptides and whole antigens. However, one healthy individual is mis-placed in the SLE group by this approach, reducing the specificity of this analysis.

The high sensitivity afforded by NIR-FE render integrated peptide and antigen arrays on plasmonic gold useful for profiling antibodies in SLE patients vs. healthy control sera. We profiled peptide interactions with antibodies in serum samples from two groups of subjects, 20 clinically confirmed SLE patients and 20 healthy controls. Serum IgG antibody reactivity against 20 unmodified and modified peptides, together with 6 whole antigens (dsDNA, U1–70, H2A, H2B, H3, H4), was obtained for each individual. For a subset of the histone peptides and most of the antigens, we found a clear increase of antibody reactivity of SLE patients compared to healthy control (see Figure 4A & Figure S3–S7). Other peptide features were either not reactive to human IgG antibodies or not specific to SLE patients, making them not specific when comparing serum from SLE patients with serum derived from the healthy control subjects (Figure 4A & Figure S3–S7). We applied Significance Analysis of Microarrays (SAM) [46] to our dataset and identified 8 histone peptides and 4 whole antigens that were significantly more reactive to IgG antibody in the sera of SLE patients than in healthy controls (false discovery rate (q value) <0.001; Table S2). The 8 peptides included unmodified and acetylated histone H2B peptides (at lysine 5, 12, 15 or 20), unmodified histone H3 and acetylated H3 (at lysine 9 and 14) peptides and acetylated H4 peptide (at lysine 5), while the antigens were histone H2B and H3 protein, U1–70 and dsDNA (Table S2). Antibody reactivity data are presented as hierarchical clusters based on Euclidean correlation [47] (Figure 4D). The hierarchical clustered heat-map successfully placed SLE patients into close subgroups, suggesting potential diagnostic value of peptides combined with antigens for profiling SLE autoantibodies. In comparison, the heatmaps based on peptide array or antigen array alone misplaced several SLE patients into the healthy group (Figure 4B, C).

## Discussion

Various methods including high-throughput assays have been developed to investigate peptide-protein, peptide-peptide and protein-protein interactions. Previously, peptide-antibody interaction has been ascertained by ELISA [48] and RIA [49] to achieve sufficient sensitivity. ELISA and RIA, however, lack multiplex capability and are unsuited for profiling large-scale peptide-protein interactions. To achieve high-throughput peptide screening, peptide microarrays have been constructed by *in situ* synthesis onto cellulose membrane (SPOT synthesis) [15], [17] or silicon, [16] or by spotting onto planar or porous support such as glass [18] or nitrocellulose [10]. *In situ* synthesis of peptide microarrays provides a high-density, well-controlled array format for high-throughput binding event analysis. Spotting peptides onto solid substrates is fast and flexible, and additionally the quality of the peptide can be well-controlled. Due to the small molecular weight and variable binding efficiency of peptides, [25] it is desired to use functionalized peptide spotted onto derivatized substrates for efficient immobilization via chemical reactions or strong biotin-streptavidin binding, as opposed to physical entrapment. Peptide arrays have also been constructed on continuous gold films for surface plasmon resonance (SPR) imaging and detection [50].

By introducing plasmonic enhanced NIR fluorescence into microarrays, we obtain a new platform for high-throughput screening and probing of biomolecular interactions with high sensitivity. Plasmonic gold film coating on glass is a simple process that affords hundreds of fold NIR fluorescence enhancement. The gold platform is compatible with existing instrumentation used for microarray experiments including printing/arraying and fluorescence scanning. The method of coating plasmonic gold substrates with an avidin layer for immobilization of biotinylated peptides is simple, effective and can be easily performed. The plasmonic microarray platform allows the use of highly diluted serum samples (up to hundreds of fold), useful when sample volumes are limited.

There are two fundamental aspects of the highly sensitive peptide microarray platform on gold. The first lies in the physics of plasmonic enhancement of NIR fluorescence by the gold nanostructures, [36], [37], [51] which boosts the signals of specific peptide-protein interactions at positive peptide-antibody binding sites. The hundreds of fold enhanced fluorescence allows for detection of fewer molecules over background noise than on traditional substrates, extending the lower limit of detection. The NIR fluorescence enhancement on plasmonic gold substrates is due to amplified excitation electric fields between metallic nanogaps, and increased radiative decay rates of excited states resulted from resonance coupling between fluorescent emission dipoles and surface plasmonic modes in gold nano-islands with an average size in the 100–200 nm range [51]. The same gold film gives little enhancement to the Cy3 dye with a fluorescence emission peak of ∼ 570 nm, well off from the NIR plasmonic modes of the Au film [32]. The second aspect is the minimal background noise and non-specific signals. We observed little autofluorescence on the gold substrate (opposite to nitrocellulose). We also developed effective chemical blocking of avidin/Au substrate through biotinylated PEG stars, which minimizes non-specific binding of abundant human antibodies in the serum, thus reducing background non-specific signals and allowing the detection of specific antibodies over a broad dynamic range down to very low concentrations.

Systemic lupus erythematosus (SLE) is the second most common rheumatic autoimmune disorder affecting 300,000 individuals in the United States, with an annual incidence of 3 cases per 100,000 persons and prevalence of 51 per 100,000 persons [52]. SLE pathogenesis is characterized by the production of serum antibodies targeting self-nuclear and cytoplasmic antigens, forming immune complexes that deposit in tissues and affect the skin, joints, lungs, kidneys, vasculature and central nervous system [53]. Recently, the Systemic Lupus International Collaborating Clinics (SLICC) group has revised and validated the American College of Rheumatology SLE criteria where seventeen criteria were proposed, including clinical criterion and immunologic criterion (i.e malar rash, oral ulcers, serositis, positive anti-nuclear antigen ANA, anti-dsDNA, etc.) be identified during any interval of observation [52]. However, despite these strict criteria, SLE is commonly misdiagnosed and mistreated, even by expert physicians [54]. The lack of validated autoantigen biomarkers to diagnosis SLE has prompted investigators to examine additional immunological features through traditional microarray on glass/nitrocellulose slides [55]–[59] to address this unmet need, including autoantibodies targeting self-peptides [54].

Our peptide-antigen microarrays on plasmonic gold substrates allow for simultaneous profiling of autoantibodies against modified and unmodified histone peptides and whole antigens, using serum samples derived from SLE patients. Antibody profiling on plasmonic gold substrate was much more sensitive with higher signal to background ratios and broader dynamic range than on glass (Figure 3), allowing for integrated peptide and antigen arrays on the same chip despite generally low peptide-antibody binding affinity compared to antigen-antibody. We found that unmodified H2B and H3 peptides composed of the tail region of histones and several of their acetylated versions are promising biomarkers for SLE. Specifically, histone H2B N-terminal peptides composed of 21 amino acids with the 5^th^, 12^th^, 15^th^ or 20^th^ lysine acetylated are reactive to IgG antibody in SLE patients, and the reactivity is higher than that of the unmodified peptide to various degrees (Figure 4B & Figure S2). This was in accord with previous ELISA measurement revealing enhanced reactivity of acetylated histone peptides (e.g., histone H2B peptide with the 12^th^ lysine acetylated) with autoantibodies in SLE patients [41], [44].

Previous histone peptide microarray on streptavidin-glass slide identified several peptides capable of differentiating SLE patient sera and healthy patient sera, including unmodified histone H3 and H2B peptide and H2B peptide with the 5^th^, 12^th^, 15^th^, 20^th^ lysine acetylated, [45] which was consistent with our data on glass (data not shown). Notably, the high sensitivity of peptide arrays on plasmonic gold led to identification of a SLE peptide marker not identified on glass, i.e., H3 (K9, 14)Ac (Figure 4B, q value <0.001, table S2). This peptide reacted with antibodies in a subset of SLE sera but gave lower signals than the acetylated H2B peptides, suggesting reactivity against an antibody in low abundance or with low affinity. No antibody reactivity towards the H3 (K9, 14)Ac peptide was observed in any of the control healthy serum (Figure 4B). This result suggested the potential of peptide microarrays on gold for identifying biomarkers against low abundance and low affinity proteins specific to a disease.

We observed highly variable IgG antibody reactivity with the histone peptides and whole histone antigens in the sera of the SLE patient group (Figure 4D). Some of the SLE sera showed high IgG antibody reactivity against a majority of the acetylated histone H2B peptides and whole histone antigens (Figure 4D, left columns). For other SLE sera, weaker IgG antibodies reactivity was observed. Patient sera may show strong reactivity against histone H2B peptides acetylated at different locations (Figure 4D, 2^nd^ and 5^th^ column) or strong reactivity with only a single acetylated H2B peptide (Figure 4D, H2B K15Ac in 4^th^ column and H2B K12Ac in 8^th^ column). We also noticed that some of the SLE sera exhibited obvious reactivity with acetylated H2B peptides (e.g., H2B K20Ac 8^th^ row; H2B K15Ac, K12Ac and K5Ac, 1^st^, 2^nd^ and 3^rd^ rows) but with little reactivity to whole H2B histone antigen (9^th^ row) or unmodified H2B peptide (6^th^ row). This is consistent with previous finding of apoptosis-induced acetylation of histone peptides in the pathogenesis of SLE [41], [44].

We found that collectively a subset of unmodified and post-translationally acetylated histone peptides in combination with several whole antigens (dsDNA, U1–70, H2B and H3) could be used to profile serum antibodies and differentiate SLE patients from healthy individuals (Figure 4D) with higher accuracy than antibody profiling with peptides alone (Figure 4B) or whole antigens alone (Figure 4C). The peptide array data alone misplaced several SLE patients in the healthy group (Figure 4B) due to low antibody reactivity with all of the peptide features. However, these patients were correctly assigned to the SLE group due to the simultaneous measured high activity against whole antigens, in particular to dsDNA. The antigen only array data misplaced several SLE patients to the healthy group due to low antibody reactivity against the four antigens tested (Figure 4C), but the simultaneously measured activity against acetylated histone peptides led to the assignment of the individuals to the SLE patient group (Figure 4D). These results suggested the potential of SLE diagnosis with high sensitivity and specificity by multiplexed antibody profiling with integrated peptide-antigen arrays on the plasmonic gold platform.

In conclusion, we have developed a new peptide-antigen microarray platform on plasmonic gold substrate capable of enhanced NIR fluorescence and background minimization. A model histone peptide microarray afforded hundreds fold NIR fluorescence enhancement and 3 orders of magnitude improvement in sensitivity and dynamic range over commercial streptavidin-glass based peptide arrays. The gold microarray platform is useful for profiling antibodies in human samples to identify peptide and antigen biomarkers, especially those capable of differentiating low abundance or affinity antibodies in SLE patient sera vs. healthy sera. The broad dynamic range allows for integrated peptide and antigen array on the same plasmonic gold chip, affording a powerful approach to profiling human antibodies for better differentiation of disease patients from healthy individuals. Notably, the peptide-antigen/gold platform can be easily adopted for use with existing microarray systems, with little change in procedures and reagents.

## Methods

### Materials

Superfrost Plus glass slides and quartz slides were purchased from Fisher Scientific and cleaned with acetone, isopropanol (IPA), and methanol. Avidin, chloroauric acid trihydrate, hydroxylamine HCl, sodium borohydride, cysteamine, 1-ethyl-3-(3 dimethylaminopropyl) carbodiimide (EDC), and *N*-hydroxysuccinimide (NHS) were purchased from Sigma–Aldrich. Ammonium hydroxide (30% ammonia) and hyclone fetal bovine serum were purchased from Fisher Chemicals. Streptavidin coated glass slides were purchased from Arrayit. Unmodified streptavidin was purchased from Jackson Immunoresearch. IRDye800-NHS ester was purchased from Licor Biosciences. 6-armed poly(ethylene glycol)–amine was purchased from SunBio, Korea. Unmodified and modified biotin conjugated peptides were ordered from Keck Biotechnology Resource Laboratory. Goat anti human IgG antibody was purchased from Vector Lab. Histone protein was purchased from Immunovision. Monoclonal mouse anti H2B K20Ac, and H3 K18Ac antibody was purchased from Abcam. Histone antigens and dsDNA were purchased from ImmunoVision. U1–70 was purchased from CPC scientific.

SLE patients and control serum samples were obtained under Stanford University Institutional Review Board approved protocols and with informed consent (Protocol #14734). Written consent was provided by participants in this study.

### Preparation of avidin coated plasmonic gold film on glass slides

Plasmonic gold substrates were synthesized by immersing glass slides into a solution of 3 mM HAuCl_4_ followed by adding ammonium hydroxide at 20 μL ammonium hydroxide per mL of HAuCl4 solution with rapid shaking for one minute. The slide was washed twice with DI water to remove unbound gold ions and immersed into 1 mM NaBH_4_ to reduce gold clusters on the glass slide to gold nanoparticle seeds. After further washing twice with water, the slides were incubated in a solution of HAuCl_4_ and hydroxylamine at a 1∶1 ratio at 750 μM each and uniformly shaken for 5 min. The slide was then soaked in 1 μM avidin in PBS solution at 4°C overnight, rinsed with PBS and then water.

### Microscopy and spectroscopy characterization of plasmonic substrate and avidin coated plasmonic substrate

Scanning electron micrographs were acquired on an FEI XL30 Sirion SEM with FEG source at 5 kV acceleration voltage. XPS was performed with a PHI VersaProbe Scanning XPS Microprobe.

### Conjugation of biotin to branched PEG-amine

Biotin-NHS ester was dissolved in dry dimethyl sulfoxide (DMSO), then mixed with branched PEG-amine in PBS at a 1∶1 mole ratio and incubated at room temperature for 2 h following excess biotin removal with a G-25 NAP-5 columns (GE healthcare).

### Peptide microarray printing and antibody detection in spiked serum

The avidin coated gold slides (or commercial streptavidin coated glass slides) were loaded into a microarray printing robot (Bio-Rad) where 0.2 mg/ml biotin conjugated peptide in PBS supplemented with 4% glycerol was printed using solid pins (Arrayit) at 25°C and 60% humidity, resulting in microarray feature diameters of ∼200 μm. The microarray layout is designed by the microarray printer software. A 4-plex peptide array was constructed in a 4×5 matrix with one peptide on each row in replicates of 5 (Figure 2A). The slides were dried in a desiccator and then blocked in 200 μM biotin conjugated PEG-star for 20 min, followed by washing twice with PBST and once with PBS. The microarray was probed with a mixture of two commercial rabbit IgG antibodies specific to the acetylated version of the two histone peptides. 20 μL of each antibody solution with concentrations from 1 nM to 10 fM in PBS with 20% FBS was applied to each set of spots along with a blank control composed of 20% FBS in PBS. After 2 h incubation at room temperature, the arrays were washed twice with PBST and once with PBS. The array was subsequently detected with 1 nM IRDye800 labeled goat IgG antibody in PBS with 20% FBS for 30 min in dark, followed by washing twice with PBST, once with PBS, once with deionized water and drying with compressed air. Identical arrays on commercial streptavidin-coated slides were also constructed and probed side by side with avidin coated plasmonic Au substrate for comparison.

### Peptide-antigen microarray for profiling of autoantibodies in human sera

Peptide and antigen printing and blocking procedure was the same as above. 20 μL of 1∶200 dilution of human serum in PBS solution with 20% FBS was applied to the array for 2 h, followed by washing twice with PBST, once with PBS. 1 nM IRDye800 labeled goat anti human IgG in PBS solution with 20% FBS was applied to each array set subsequently for 20 min in dark. After that, the slide was washed twice with PBST, once with PBS, once with deionized water and dried with compressed air.

### Fluorescence measurement and SAM analysis

The commercial Licor Odyssey scanner was applied to scan the peptide microarray assays on different substrates with the 800 nm channel. Genepix 6.1 was used to automatically identify features above composite pixel intensity of 5. Fluorescence intensity for each set of features was background corrected average of mean pixel intensity values for features printed in replicates. Multi-experiment viewer (MEV TM4 Microarray Software Suite version 10.2, Dana-Farber Cancer Institute, Boston, MA) was used to implement significance analysis of microarray for the human sera profiling result based on multiplexed peptide microarray. The SAM algorithm was applied to numerical MFI (mean fluorescence intensity) values. Peptide and antigen reactivity that significantly correlated with SLE (vs. healthy control) classes was determined by 1,000 permutations of repeated measurements to have a false discovery rate of 0 (q value <0.001). Autoantibody reactivity heatmaps were generated using Multiexperiment Viewer using average linkage Euclidean distance hierarchical clustering.

## Supporting Information

Figure S1 **Fluorescence enhancement dependence on the number of protein layers.** A) For the ‘avidin IR800’ column: Mean IRDye800 fluorescence signal for a monolayer of IRDye800 labeled avidin adsorbed on plasmonic film and glass slide. Both the plasmonic film and glass slide were soaked in an IRDye800 labeled avidin solution at 4°C overnight and then rinsed with water prior to fluorescence intensity measurement with a Licor Odyssey scanner. For the ‘avidin-biotin BSA-avidin IR800’ column: signals measured on 3 layers of avidin – biotinylated BSA – IRDye800 labeled avidin on plasmonic film and glass slide respectively. Both the plasmonic film and glass slide were soaked in avidin at 4°C overnight, after washing with twice PBST and once PBS, the slides was soaked in biotin conjugated BSA solution for 1h, followed by washing with PBST twice and PBS once and incubation in IRDye800 labeled avidin for 1h. Fluorescence intensity was checked by Licor Odyssey scanner. In this case, the IR800 labeled avidin is on the third layer of the protein stack. B) Fluorescence enhancement factor (signal on gold divided by signal on glass) for 1 layer and 3 layer structures based on the data in A), suggesting no significant fluorescence enhancement loss when fluorophore is two protein layer away from the plasmonic substrate.
(TIF)

Figure S2 **Peptide microarray profiling with different blocking reagents.** The avidin coated gold slides were loaded into a microarray printing robot (Bio-Rad) where 0.2 mg/ml biotin conjugated peptide H2B K12Ac, H3 K18Me1, H2B K5Ac, H2B (aa1–21) were printed in 4 rows with triplicates. The slides were dried in a desiccator and then blocked in 200 μM biotin conjugated PEG-star, biotin conjugated straight PEG chain or biotin only for 20 min, followed by washing twice with PBST and once with PBS. The microarray was probed with SLE serum sample and detected with IRDye800 labeled antihuman IgG antibody. A) Fluorescence images for SLE patient serum probed on avidin/gold slide with biotin-PEG star blocking, linear biotin-PEG blocking, and biotin blocking respectively. B) Corresponding background and spot signals for the three blocking methods in (A). The lowest background was detected with biotin-PEG stars, which facilitated higher signal/noise ratios and peptide arrays with high sensitivity and broad dynamic ranges.
(TIF)

Figure S3 **Box plot of serum IgG antibody reactivity from 20 SLE patients and 20 healthy controls against unmodified and modified histone H2A peptides.**
(TIF)

Figure S4 **Box plot of serum IgG antibody reactivity from 20 SLE patients and 20 healthy controls against unmodified and modified histone H2B peptides.**
(TIF)

Figure S5 **Box plot of serum IgG antibody reactivity from 20 SLE patients and 20 healthy controls against unmodified and modified histone H3 peptides.**
(TIF)

Figure S6 **Box plot of serum IgG antibody reactivity from 20 SLE patients and 20 healthy controls against unmodified and modified histone H4 peptides.**
(TIF)

Figure S7 **Box plot for SLE patient and healthy control sera IgG antibody reactivity against whole antigens including histone H2A, H2B, H3 and H4 proteins, U1–70 and dsDNA.**
(TIF)

Table S1 **Amino acid sequences of printed histone peptides in the peptide-antigen arrays.** Ac: acetylated; aa: amino acid; Me1: methylated; Me2: dimethylated; Me3: trimethylated; Ph: phosphorylated; K: Lysine; S: Serine. Number indicates amino acid position from the N-terminus of its corresponding histone proteins.
(TIF)

Table S2 **q- and p-values for peptides and antigens included in the peptide-antigen microarray platform in differentiating SLE patient and healthy control groups derived from Significance Analysis of Microarray (SAM).**
(TIF)
