# Supplementary figures and images for: An Integrated Peptide-Antigen Microarray on Plasmonic Gold Films for Sensitive Human Antibody Profiling
Source: PLoS One. 2013 Jul 29;8(7):e71043. doi: 10.1371/journal.pone.0071043 (PMC3726620; doi:10.1371/journal.pone.0071043)

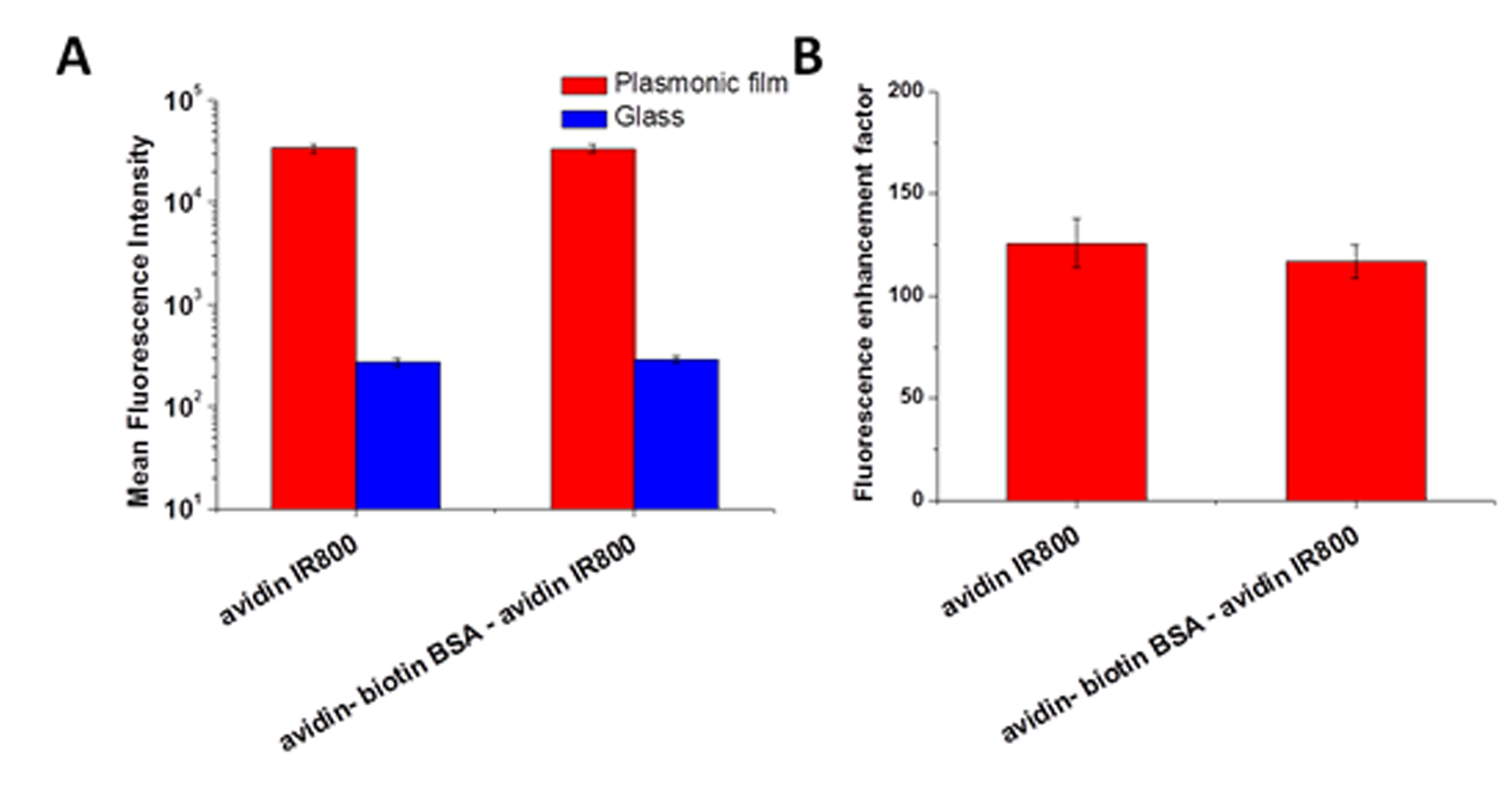

Supplement: Figure S1 — Fluorescence enhancement dependence on the number of protein layers. A) For the ‘avidin IR800’ column: Mean IRDye800 fluorescence signal for a monolayer of IRDye800 labeled avidin adsorbed on plasmonic film and glass slide. Both the plasmonic film and glass slide were soaked in an IRDye800 labeled avidin solution at 4°C overnight and then rinsed with water prior to fluorescence intensity measurement with a Licor Odyssey scanner. For the ‘avidin-biotin BSA-avidin IR800’ column: signals measured on 3 layers of avidin – biotinylated BSA – IRDye800 labeled avidin on plasmonic film and glass slide respectively. Both the plasmonic film and glass slide were soaked in avidin at 4°C overnight, after washing with twice PBST and once PBS, the slides was soaked in biotin conjugated BSA solution for 1h, followed by washing with PBST twice and PBS once and incubation in IRDye800 labeled avidin for 1h. Fluorescence intensity was checked by Licor Odyssey scanner. In this case, the IR800 labeled avidin is on the third layer of the protein stack. B) Fluorescence enhancement factor (signal on gold divided by signal on glass) for 1 layer and 3 layer structures based on the data in A), suggesting no significant fluorescence enhancement loss when fluorophore is two protein layer away from the plasmonic substrate. (TIF) [file pone.0071043.s001.tif]

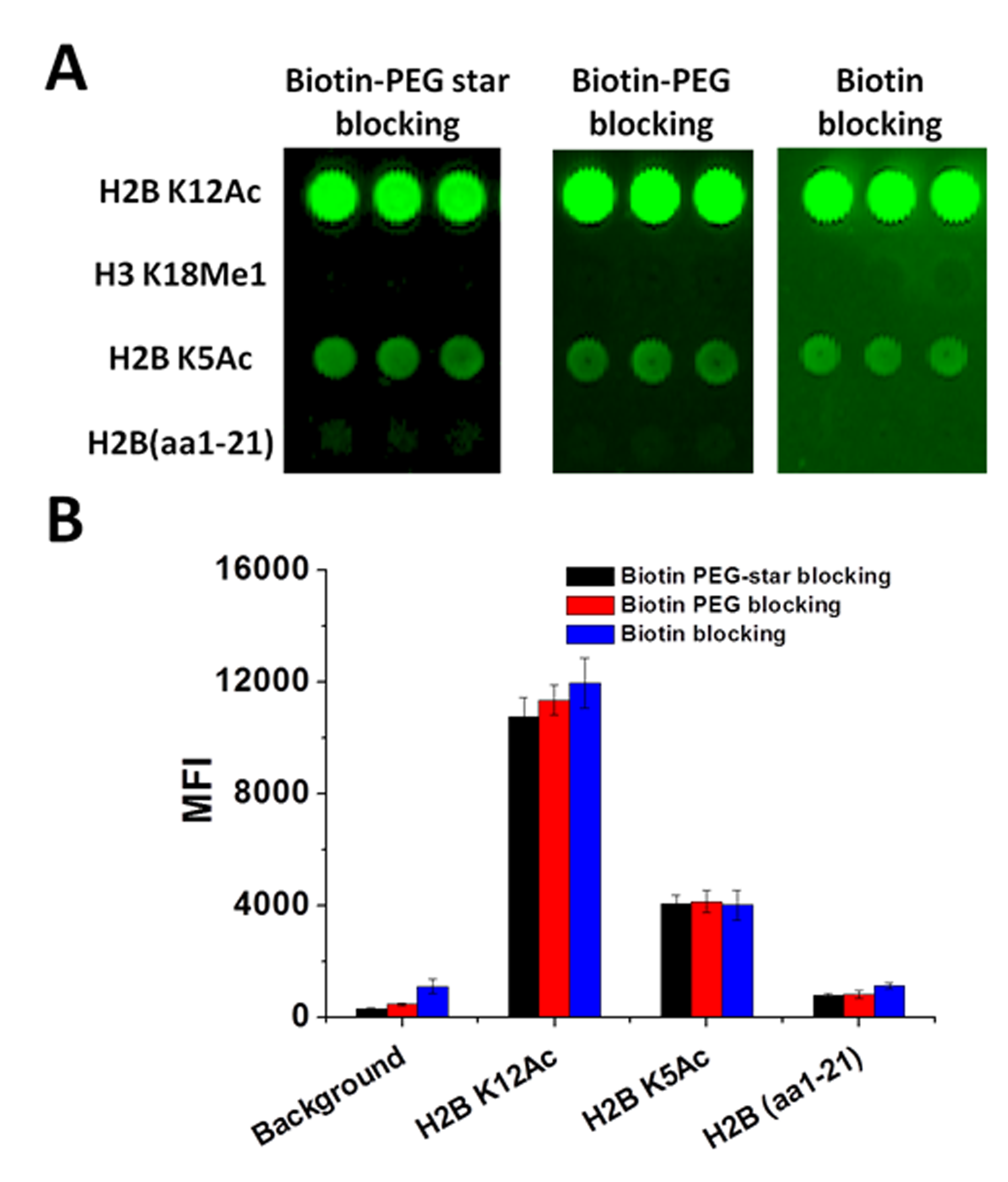

Supplement: Figure S2 — Peptide microarray profiling with different blocking reagents. The avidin coated gold slides were loaded into a microarray printing robot (Bio-Rad) where 0.2 mg/ml biotin conjugated peptide H2B K12Ac, H3 K18Me1, H2B K5Ac, H2B (aa1–21) were printed in 4 rows with triplicates. The slides were dried in a desiccator and then blocked in 200 μM biotin conjugated PEG-star, biotin conjugated straight PEG chain or biotin only for 20 min, followed by washing twice with PBST and once with PBS. The microarray was probed with SLE serum sample and detected with IRDye800 labeled antihuman IgG antibody. A) Fluorescence images for SLE patient serum probed on avidin/gold slide with biotin-PEG star blocking, linear biotin-PEG blocking, and biotin blocking respectively. B) Corresponding background and spot signals for the three blocking methods in (A). The lowest background was detected with biotin-PEG stars, which facilitated higher signal/noise ratios and peptide arrays with high sensitivity and broad dynamic ranges. (TIF) [file pone.0071043.s002.tif]

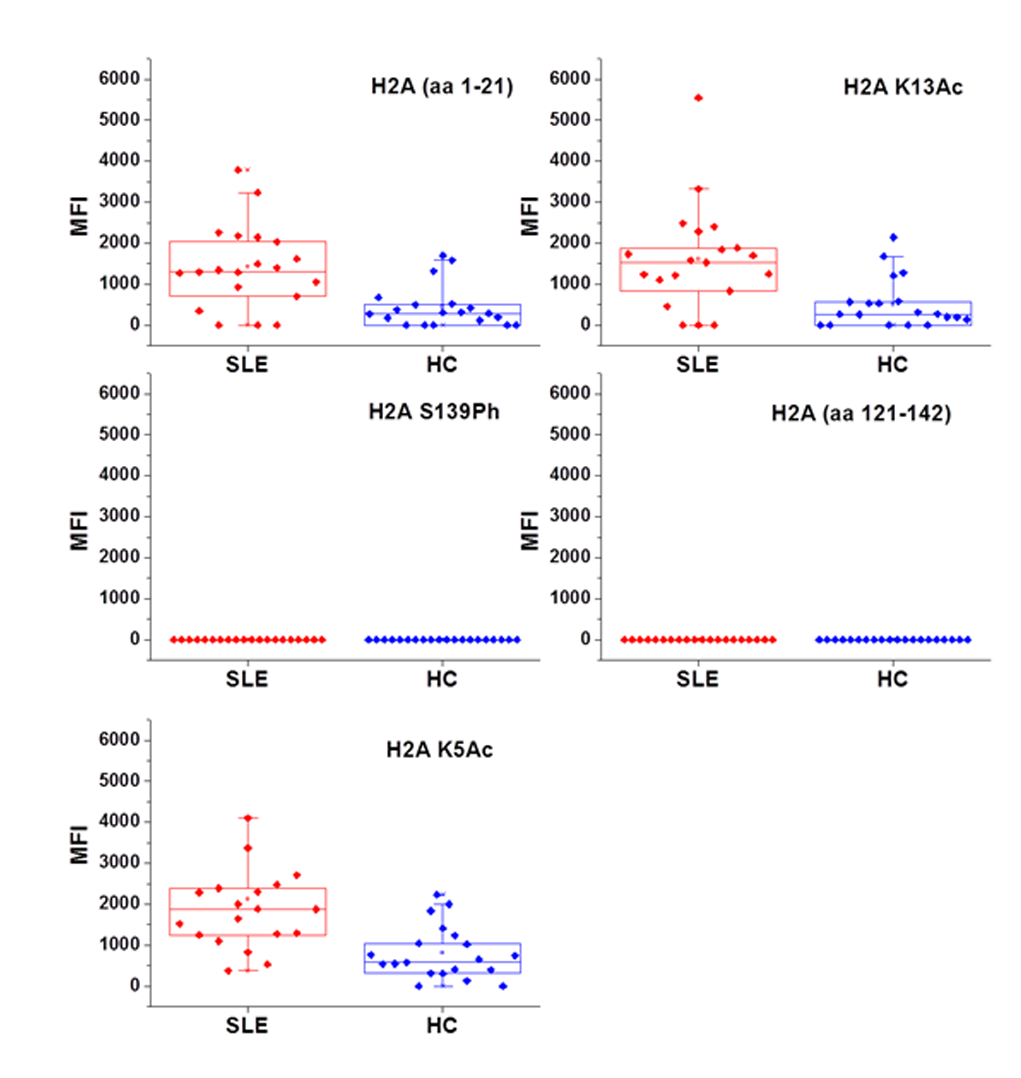

Supplement: Figure S3 — Box plot of serum IgG antibody reactivity from 20 SLE patients and 20 healthy controls against unmodified and modified histone H2A peptides. (TIF) [file pone.0071043.s003.tif]

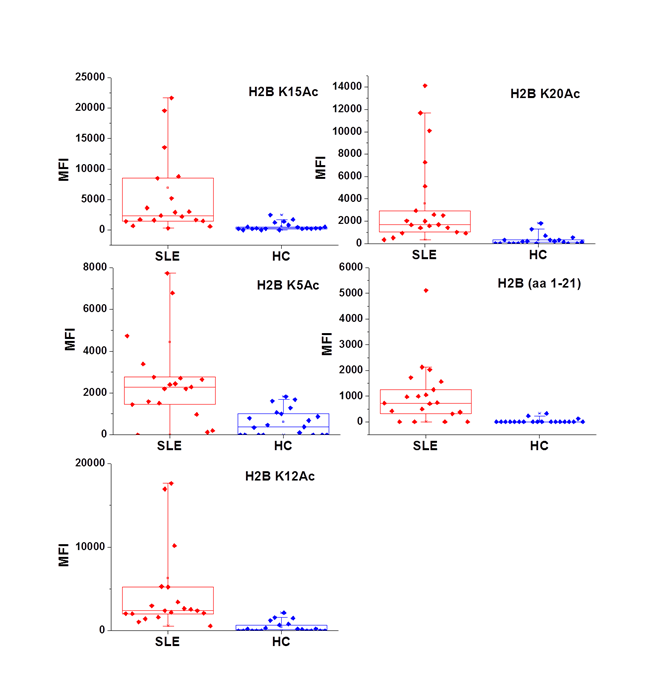

Supplement: Figure S4 — Box plot of serum IgG antibody reactivity from 20 SLE patients and 20 healthy controls against unmodified and modified histone H2B peptides. (TIF) [file pone.0071043.s004.tif]

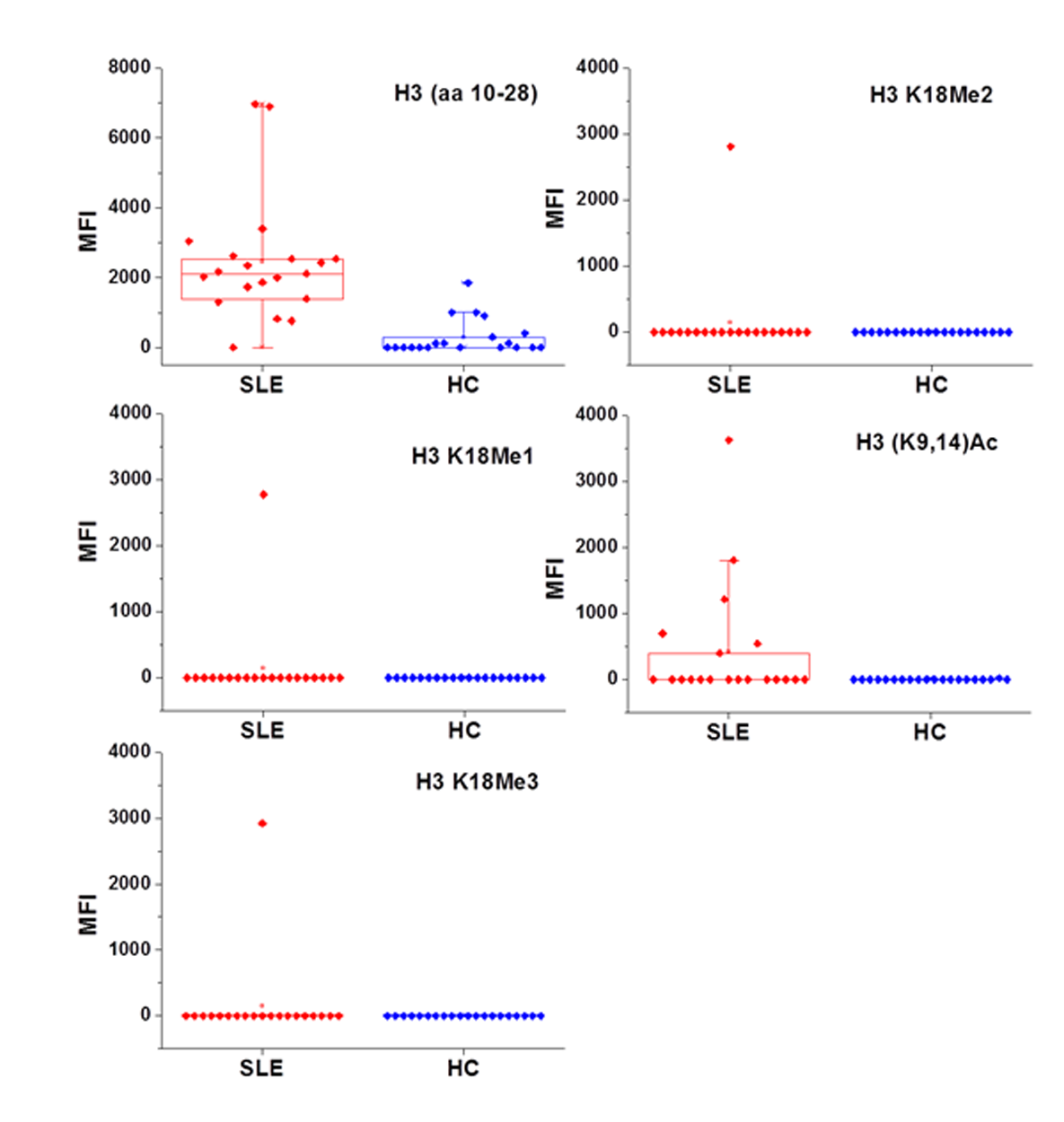

Supplement: Figure S5 — Box plot of serum IgG antibody reactivity from 20 SLE patients and 20 healthy controls against unmodified and modified histone H3 peptides. (TIF) [file pone.0071043.s005.tif]

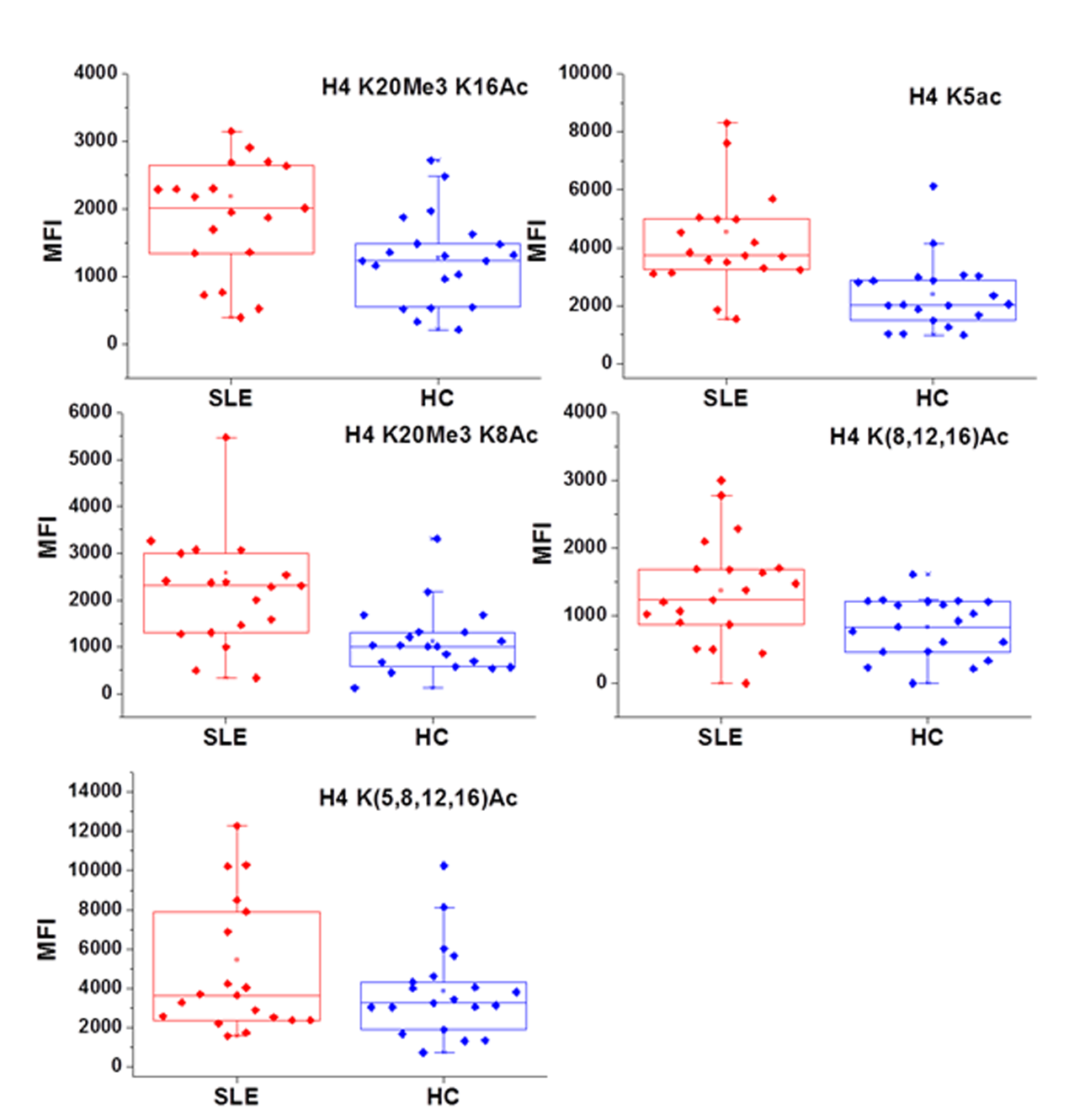

Supplement: Figure S6 — Box plot of serum IgG antibody reactivity from 20 SLE patients and 20 healthy controls against unmodified and modified histone H4 peptides. (TIF) [file pone.0071043.s006.tif]

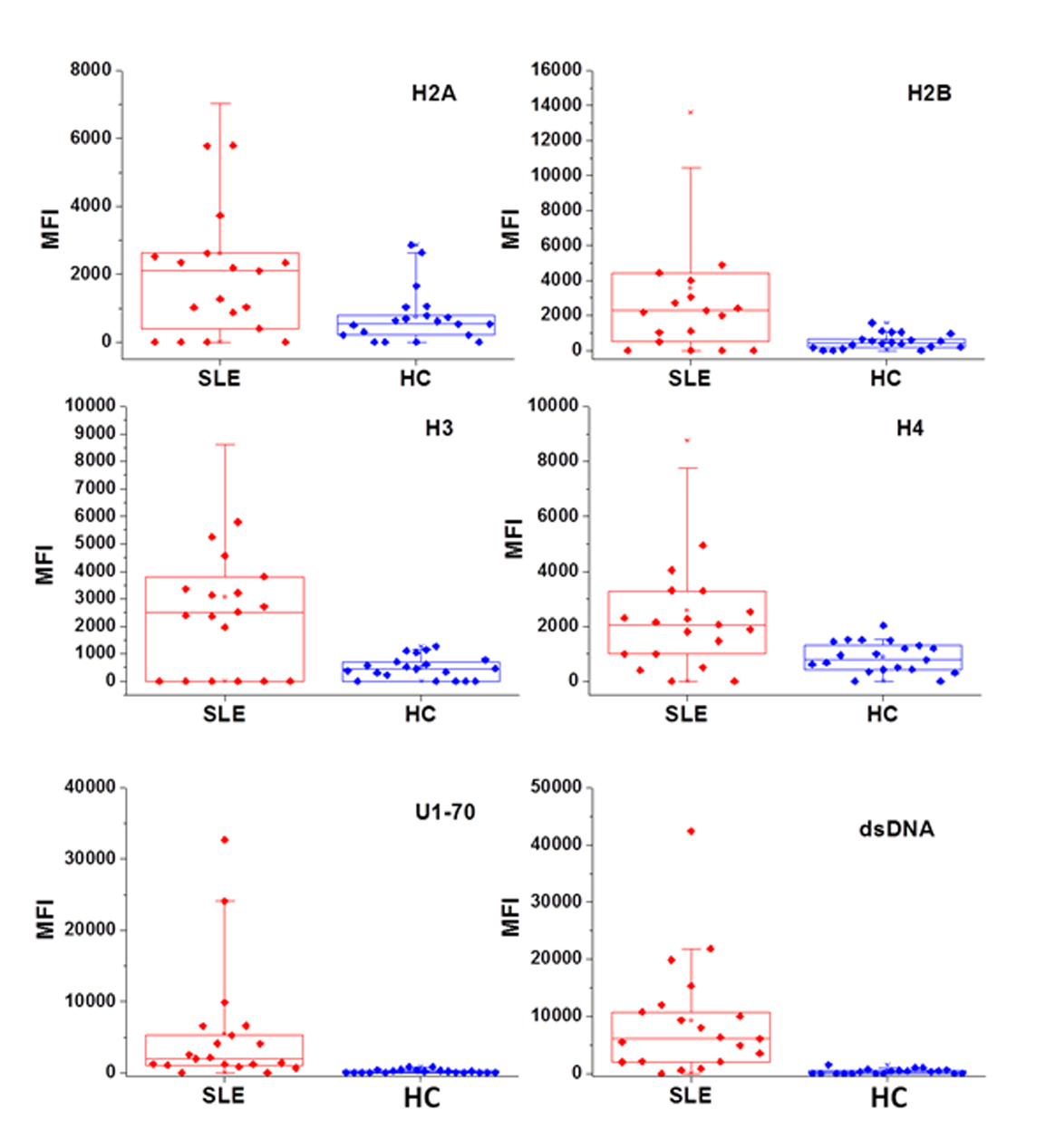

Supplement: Figure S7 — Box plot for SLE patient and healthy control sera IgG antibody reactivity against whole antigens including histone H2A, H2B, H3 and H4 proteins, U1–70 and dsDNA. (TIF) [file pone.0071043.s007.tif]

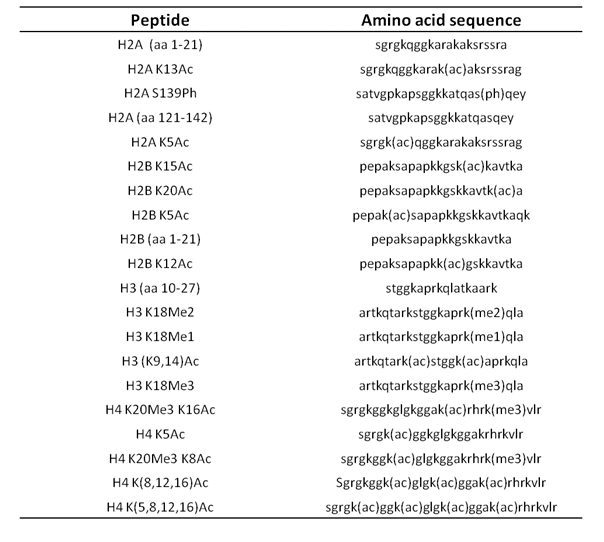

Supplement: Table S1 — Amino acid sequences of printed histone peptides in the peptide-antigen arrays. Ac: acetylated; aa: amino acid; Me1: methylated; Me2: dimethylated; Me3: trimethylated; Ph: phosphorylated; K: Lysine; S: Serine. Number indicates amino acid position from the N-terminus of its corresponding histone proteins. (TIF) [file pone.0071043.s008.tif]

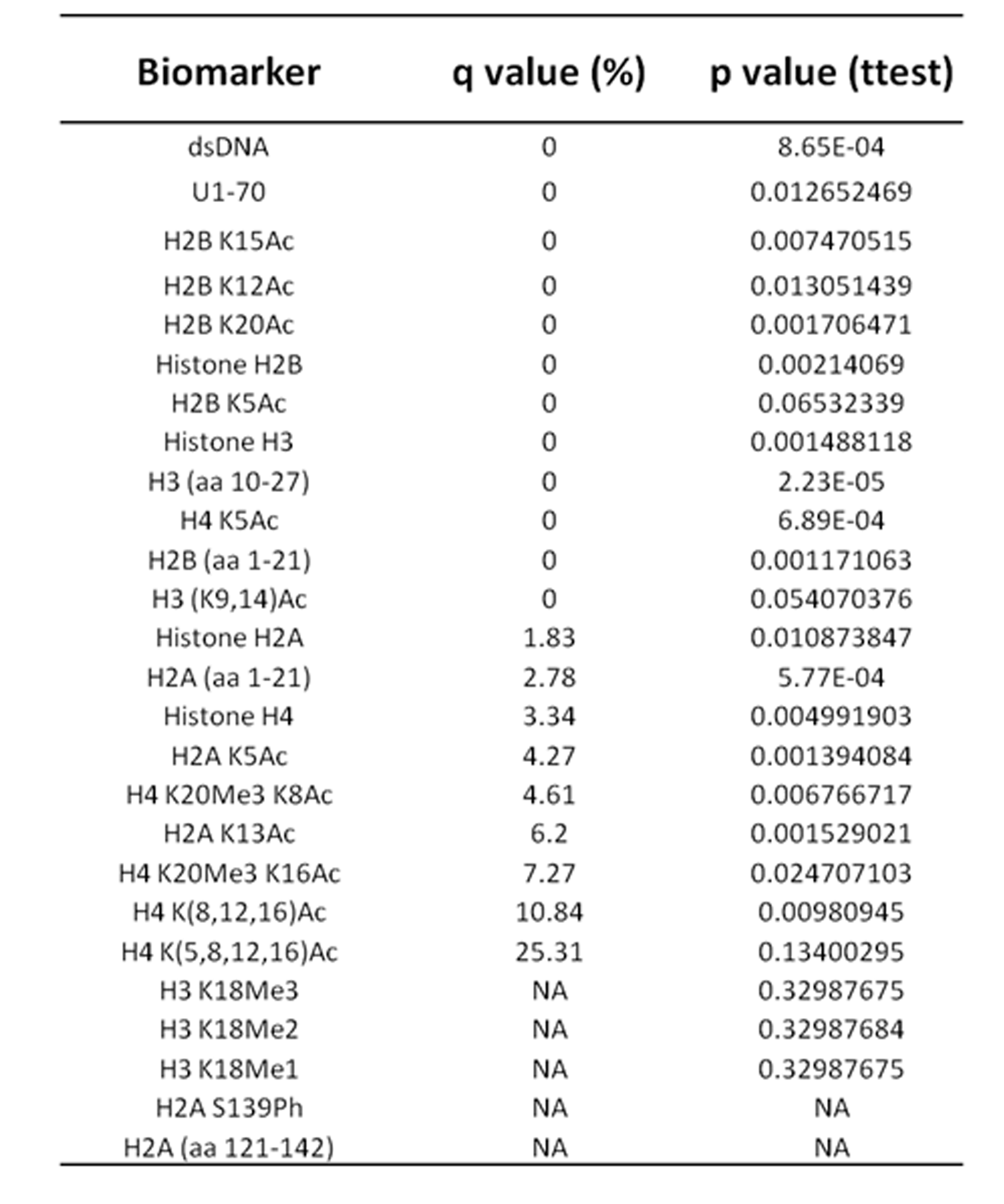

Supplement: Table S2 — q- and p-values for peptides and antigens included in the peptide-antigen microarray platform in differentiating SLE patient and healthy control groups derived from Significance Analysis of Microarray (SAM). (TIF) [file pone.0071043.s009.tif]
